# Spontaneous Differentiation of Human Mesenchymal Stem Cells on Poly-Lactic-Co-Glycolic Acid Nano-Fiber Scaffold

**DOI:** 10.1371/journal.pone.0153231

**Published:** 2016-04-07

**Authors:** Koshiro Sonomoto, Kunihiro Yamaoka, Hiroaki Kaneko, Kaoru Yamagata, Kei Sakata, Xiangmei Zhang, Masahiro Kondo, Yukichi Zenke, Ken Sabanai, Shingo Nakayamada, Akinori Sakai, Yoshiya Tanaka

**Affiliations:** 1 The First department of internal medicine, school of medicine, University of occupational and environmental health, Kitakyushu, Fukuoka, Japan; 2 Integrative technology research institute, Teijin Limited, Hino, Tokyo, Japan; 3 Department of orthopedics, school of medicine, University of occupational and environmental health, Kitakyushu, Fukuoka, Japan; 4 Rheumatology, National hospital organization Beppu medical center, Beppu, Oita, Japan; 5 Internal medicine, division of rheumatology, Keio university, Shinjuku-ku, Tokyo, Japan; 6 Research unit B, research division, Mitsubishi Tanabe pharma corporation, Yokohama, Kanagawa, Japan; University of Torino, ITALY

## Abstract

**Introduction:**

Mesenchymal stem cells (MSCs) have immunosuppressive activity and can differentiate into bone and cartilage; and thus seem ideal for treatment of rheumatoid arthritis (RA). Here, we investigated the osteogenesis and chondrogenesis potentials of MSCs seeded onto nano-fiber scaffolds (NFs) *in vitro* and possible use for the repair of RA-affected joints.

**Methods:**

MSCs derived from healthy donors and patients with RA or osteoarthritis (OA) were seeded on poly-lactic-glycolic acid (PLGA) electrospun NFs and cultured *in vitro*.

**Results:**

Healthy donor-derived MSCs seeded onto NFs stained positive with von Kossa at Day 14 post-stimulation for osteoblast differentiation. Similarly, MSCs stained positive with Safranin O at Day 14 post-stimulation for chondrocyte differentiation. Surprisingly, even cultured without any stimulation, MSCs expressed RUNX2 and SOX9 (master regulators of bone and cartilage differentiation) at Day 7. Moreover, MSCs stained positive for osteocalcin, a bone marker, and simultaneously also with Safranin O at Day 14. On Day 28, the cell morphology changed from a spindle-like to an osteocyte-like appearance with processes, along with the expression of dentin matrix protein-1 (DMP-1) and matrix extracellular phosphoglycoprotein (MEPE), suggesting possible differentiation of MSCs into osteocytes. Calcification was observed on Day 56. Expression of osteoblast and chondrocyte differentiation markers was also noted in MSCs derived from RA or OA patients seeded on NFs. Lactic acid present in NFs potentially induced MSC differentiation into osteoblasts.

**Conclusions:**

Our PLGA scaffold NFs induced MSC differentiation into bone and cartilage. NFs induction process resembled the procedure of endochondral ossification. This finding indicates that the combination of MSCs and NFs is a promising therapeutic technique for the repair of RA or OA joints affected by bone and cartilage destruction.

## Introduction

Rheumatoid arthritis (RA) is a systemic autoimmune disease characterized by chronic synovitis of the joints, in which cytokines such as tumor necrosis factors (TNFs) and interleukins (ILs) contribute to the pathogenesis, causing excessive bone resorption and chondral degradation, and resulting in articular deformities [1]. Joint destruction in RA patients is characterized by bone resorption by osteoclasts, which are differentiated and activated by various mediators secreted from proliferative synovial membranes, and digestion of cartilage by matrix metalloproteinases (MMPs) [1]. RA-related joint destruction leads to impaired physical function, and it is difficult to repair damaged joints with drugs or other therapeutic measures. Surgical insertion of joint prosthesis is indicated for severely damaged large joints. However, when small joints are damaged by RA, restoration of function is often difficult because of technical issues (i.e., complexity of surgery and impairment of the devices).

Mesenchymal stem cells (MSCs) are cell population that can differentiate into osteoblasts, chondrocytes, or adipocytes, with replication competence [2]. Because they are widely distributed throughout the body, including the bone marrow and subcutaneous adipose tissue, and can divide in *vitro*, MSCs are relatively easy to obtain and have been studied for various clinical applications. Horwitz et al. [3] performed allotransplantation of MSCs in patients with osteogenesis imperfecta and showed that MSCs could be applicable for the treatment of bone diseases. Furthermore, MSCs are known to mediate immunosuppression through various soluble mediators such as transforming growth factor (TGF)-beta [4], prostaglandins, inducible nitric-oxide synthase, or indole 2,3-dioxygenase [5], and Le Blank et al. [6] reported the efficacy and safety of allotransplantation of MSCs in patients with graft-versus-host disease. We hypothesized that MSCs, which can differentiate into joint component cells and have immunosuppressive activity as described above, could be used for the repair of RA-affected joints.

In RA, the receptor activator of nuclear factor-kappa B ligand (RANKL), which is expressed in proliferative synovial membrane tissue, induces differentiation of osteoclasts, leading to excessive bone resorption. We reported previously that MSCs inhibit osteoclast differentiation and function by constitutive production of osteoprotegerin (OPG), a decoy-receptor of RANKL [7]. In another study, we also reported that IL-1beta and other inflammatory cytokines activate the wingless-type MMTV integration site family (WNT) 5a/receptor tyrosine kinase-like orphan receptor (ROR) 2 pathway, which in turn induces MSCs to differentiate into osteoblasts [8]. These studies indicate that transplantation of MSCs could potentially inhibit the progression of bone destruction and simultaneously results in bone regeneration in patients with uncontrollable arthritis.

In the next series of studies, we investigated the suitability of various routes for administration of MSCs. Given our goals of controlling arthritis and regeneration of joints, local administration of MSCs appeared necessary. Although intravenous administration, as used by several groups, is simple and easy [6], intravenously transplanted MSCs may be captured by the pulmonary circulation [9]. Thus, MSCs administered through this route have less chance of reaching the affected joints. Intriguingly, the results of another study from our laboratory showed that MSCs injected directly into a joint in the form of a cell suspension disappeared immediately from the joint [4]. Next, we investigated whether the use of scaffolds could solve this problem. When MSCs were seeded on poly-lactic-co-glycolic acid (PLGA) nano-fiber scaffolds (NFs) followed by transplantation of the NFs into the ankle joints of collagen-induced arthritis (CIA) rats, the MSCs remained in the joints, resulting in the suppression of arthritis and inhibition of joint destruction. Our results also showed no MSCs in the ankle joints and no inhibitory effects on either arthritis or joint destruction in rats following intra-articular or intraperitoneal injection of MSCs alone [4]. The above study showed that the combination of MSCs and NFs may suppress arthritis and inhibit joint destruction, and that repair of affected joints can be expected after the establishment of MSCs in the joints. In fact, a previous study reported that the use of NFs identical to those used in our rat experiments resulted in regeneration of cartilage and bone in a rabbit model of chondral defect [10]. Based on the above work, the present study was designed to determine in detail the *in vitro* effects of PLGA NFs on the differentiation of MSCs.

## Materials and Methods

### Preparation of human MSCs (hMSCs)

hMSCs from healthy donors and skin fibroblasts from different healthy donors were purchased from Lonza (Walkersville, MD), plated in cell culture flasks, and expanded in mesenchymal stem cell growth medium (MSCGM; MSCGM BulletKit, Lonza) at 37°C under 5% CO_2_ atmosphere for 7–10 days. Following incubation for adequate cell growth, adherent cells were trypsinized and then used for further experiments. All healthy donors were 23–40 years old and females.

hMSCs derived from patients with RA (diagnosed according to the 2010 rheumatoid arthritis classification criteria [11]) or osteoarthritis (OA) (diagnosed according to the ACR Clinical classification criteria for Osteoarthritis of the knee [12]) were collected from patients undergoing surgery for total hip arthroplasty or total knee arthroplasty at the Department of Orthopedics, University Hospital of Occupational and Environmental Health, Japan. Bone marrow fluid was collected non-invasively during surgery. Mononuclear cells from the collected bone marrow fluid were isolated by density-gradient centrifugation, plated in culture flasks, and expanded in MSCGM. Disease-derived hMSCs were used for further experiments after two rounds of subculture. All donors were 54–79 years old and females.

The study protocols were approved by the Ethics Committee of University Hospital of Occupational and Environmental Health, Japan, and all subjects were informed of the potential benefits and risks of the treatments and of their right to withdraw from the study. All participants provided written informed consent.

### Flow cytometry

The expressions of cell surface molecules of all MSCs were analyzed before experimental use. MSCs were suspended and incubated in blocking buffer (PBS with 1% of bovine serum albumin) for 15 minutes. After centrifuging and washing, cells were stained with following antibodies for 5 minutes; anti- human CD44 PE (193–050, Ancell corporation, MA), anti-human CD105 PE-Cy7 (25-1057-42, eBioscience, CA), anti-human CD90 PerCP-Cy5.5 (45-0909-42, eBioscience) and anti-human CD73 APC (17-0739-42, eBioscience). After washing, expressions of these molecules were analyzed by FACSCalibur (BD Bioscience, CA).

### Three-dimensional culture on NFs

The preparation of poly-lactic-co-glycolic acid NFs was described in detail previously [10]. Columnar-shaped nano-fiber plugs were used in this study. The molar ratio of lactide to glycolide was 50:50. For three-dimensional cultures, 1 × 10^6^ hMSCs or skin fibroblasts were injected into the NFs and cultured in 12-well plastic plates with 2 ml of media. Osteogenic induction medium (OIM, hMSC Differentiation BulletKit, Osteogenic, Lonza) and chondrogenic induction medium (CM, hMSC Differentiation BulletKit-chondrogenic, Lonza) in the presence of 10 ng/ml recombinant human TGF-beta3 (Lonza) were used for osteogenic and chondrogenic cultures, respectively. During the experiments, the medium was changed every 2 to 3 days.

### Histology

NFs were harvested and fixed for 2 h in 10% buffered formalin at room temperature and prepared for paraffin embedding. To detect calcium deposition, sections were stained with 5% silver nitrate solution (ID Labs Biotechnology, Buffalo, NY), exposed to ultraviolet light for 30 min, and stained with nuclear fast red solution (ID Labs Biotechnology). To detect matrix proteoglycans, sections were stained with 0.1% Safranin O solution (Muto Pure Chemicals, Tokyo, Japan) and counter-stained with hematoxylin.

The primary antibodies used for immunohistochemistry included monoclonal mouse anti-human runt-related transcription factor 2 (RUNX2; H00000860-M06, Abnova, Taipei, Taiwan, 1: 100 dilution), monoclonal mouse anti-human osteocalcin (MAB1419, R&D, Minneapolis, MN, 1: 1000 dilution), monoclonal mouse anti-human dentin matrix protein-1 (DMP-1; sc-73633, Santa Cruz, Dallas, TX, 1: 100 dilution), and polyclonal rabbit anti-human type-II collagen antibody (ab34712, Abcam, Cambridge, MA, 1: 100 dilution). The secondary antibodies were horseradish peroxidase (HRP)-conjugated antibodies (Simple stain MAX PO, Nichirei, Tokyo, Japan). Antigens were visualized using a 3,3-diaminobenzidine tetrahydrochloride substrate (Dako, Glostrup, Denmark) and counter-stained with hematoxylin.

### Real-time polymerase chain reaction (PCR)

Gene expression was assessed by real-time PCR. Total RNA was extracted using the RNeasy Mini Kit (Qiagen, Hilden, Germany), according to the protocol provided by the manufacturer. The following gene-specific primers (Applied Biosystems, Foster City, CA) were used: Hs00165814_m1for SOX9, Hs00166657_m1 for type X collagen (*COL10A1*), Hs00153936_m1 for aggrecan (*AGN*), Hs01047978_m1 for RUNX2, Hs01587814_g1 for osteocalcin (bone γ-carboxyglutamic acid-containing protein; *BGLAP*), and Hs00220237_m1 for matrix extracellular phosphoglycoprotein (*MEPE*). The expression levels of the tested genes were normalized by the expression levels of a reference gene beta-actin (*ACTB*, Hs99999903_m1) and calculated using the ΔΔCt method.

### Scanning electron microscopy

NFs cultured with MSCs were fixed in 2% glutaraldehyde and 2% paraformaldehyde. In the next step, the samples were fixed in 1% OsO_4_ and dehydrated using a series of graded alcohol solutions. After freeze-drying in t-butyl-alcohol, the samples were examined under a scanning electron microscope (TM3000; Hitachi, Tokyo).

### Culture of hMSCs with lactic acid

hMSCs were seeded at a density of 5,000 cells/cm^2^ in 24-well plastic plates. After 24 h, the medium was replaced with fresh MSCGM, and l-lactic acid (Sigma-Aldrich, St. Louis, MO) was added to the culture medium (Day 0).

Cell apoptosis was assessed at Day 7 by propidium iodide (PI) staining. The cultured cells were trypsinized and suspended in PBS supplemented with 2% fetal bovine serum (FBS) and 500 ng/ml propidium iodide (PI). PI fluorescence was determined using a FACSCalibur flow cytometer (Becton Dickinson, Franklin Lakes, NJ), and PI-positive cells were counted as apoptotic cells. The number of viable cells in each well was determined by the water-soluble tetrazolium (WST) assay using the TetraColor ONE Kit (Seikagaku, Tokyo) at Day 7 post-treatment with lactic acid, using the instructions supplied by the manufacturer.

Alkaline phosphatase (ALP) activity was quantified using the LabAssay *p*-Nitrophenylphosphate Detection Kit (Wako Pure Chemical Industries, Osaka, Japan) at Day 7 post-treatment with lactic acid, according to the protocol recommended by the manufacturer.

The production of sulfated glycosaminoglycan (sGAG) was measured using the Blyscan Glycosaminoglycan Assay Kit (Biocolor, County Antrim, UK) at Day 28 post-treatment with lactic acid, according to the protocol recommended by the manufacturer.

During the experiments, the medium was changed every 2 to 3 days.

### Measurement of cell proliferation

hMSCs (2 × 10^3^) were seeded in 96-well plates and cultured for 6 days in MSCGM, with or without l-lactate. Subsequently, 0.5 μCi ^3^H-thymidine was added to each well. After 16 h, radioactivity was measured using a scintillation beta counter (TopCount NXT, Perkin Elmer, Waltham, MA).

### Statistical analysis

Data are expressed as mean ± standard error of the mean (SEM). The paired *t*-test was used to test for differences between two groups. Analysis of variance (ANOVA) was used for comparison of three or more groups, with the post-hoc Dunnett’s multiple comparison test. In all analyses, *p* values <0.05 were considered significant.

## Results

**Fig 1:**
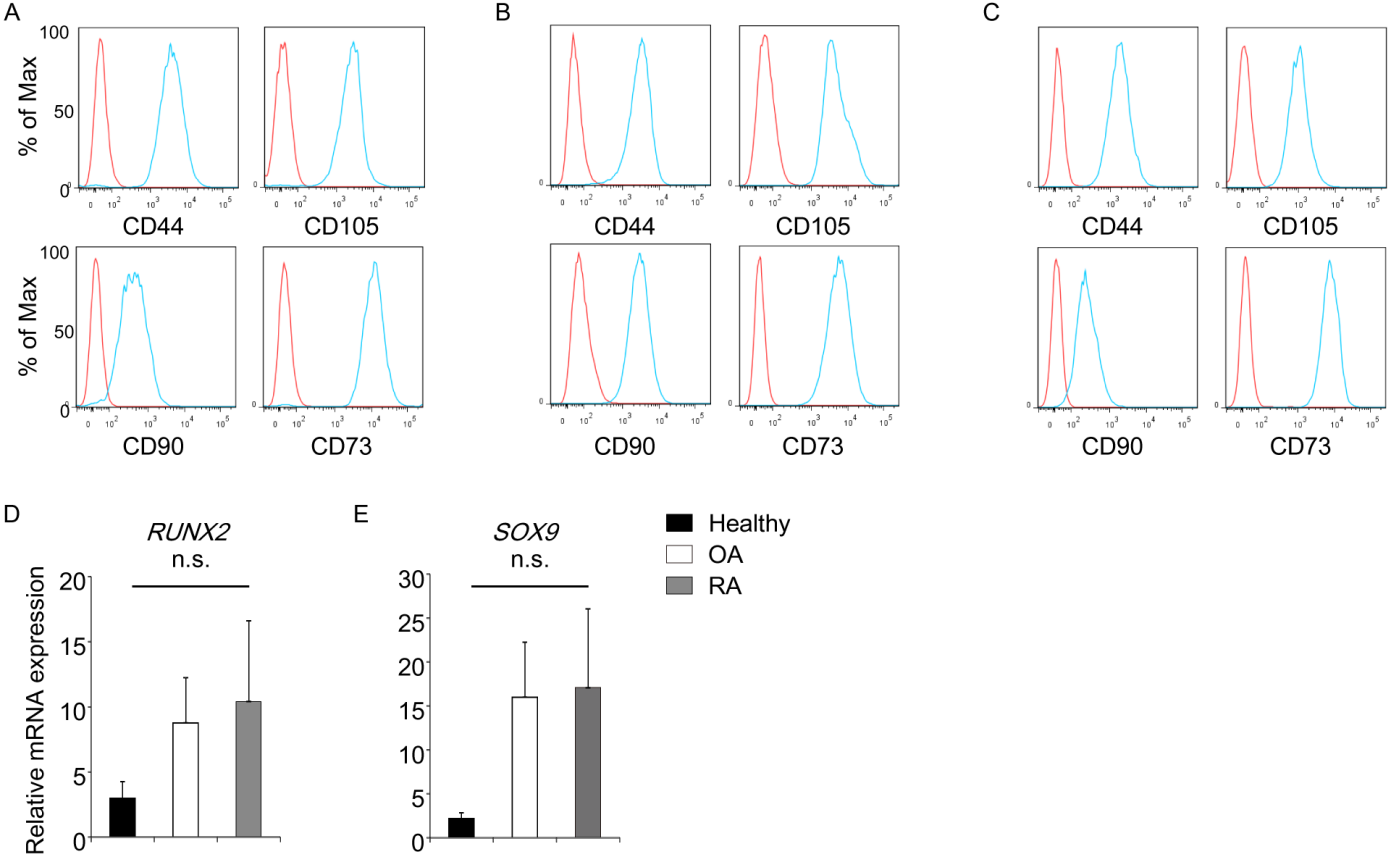
Characteristics of hMSCs derived from healthy donors or patients with RA/OA. Expanded bone marrow cells collected from healthy donors (n = 6) or patients with RA (n = 3), OA (n = 3) were analyzed as for their characteristics. Representative histograms of the cell surface markers on bone marrow cells derived from healthy donors (A), patients with RA (B), OA (C) were shown. Red lines show isotype controls. Gene expression of *RUNX2* (D) and *SOX9* (E) in hMSCs was analyzed by real-time PCR. n.s.: not significant by ANOVA.

First, we analyzed the character of the cells used for following experiments. It is well known that MSCs express CD44, CD73, CD90 and CD105 on their cell surface [13]. All bone marrow cells used in the present study expressed these molecules, which were consistent with hMSCs (Fig 1A–1C). Real-time PCR experiments were performed in order to evaluate the differentiation potential of RA- or OA-derived MSCs. Relatively higher mRNA expression of RUNX2 and SOX9 was observed in patients-derived hMSCs, however, there was no statistical difference among the cases and the healthy (Fig 1D and 1E).

**Fig 2:**
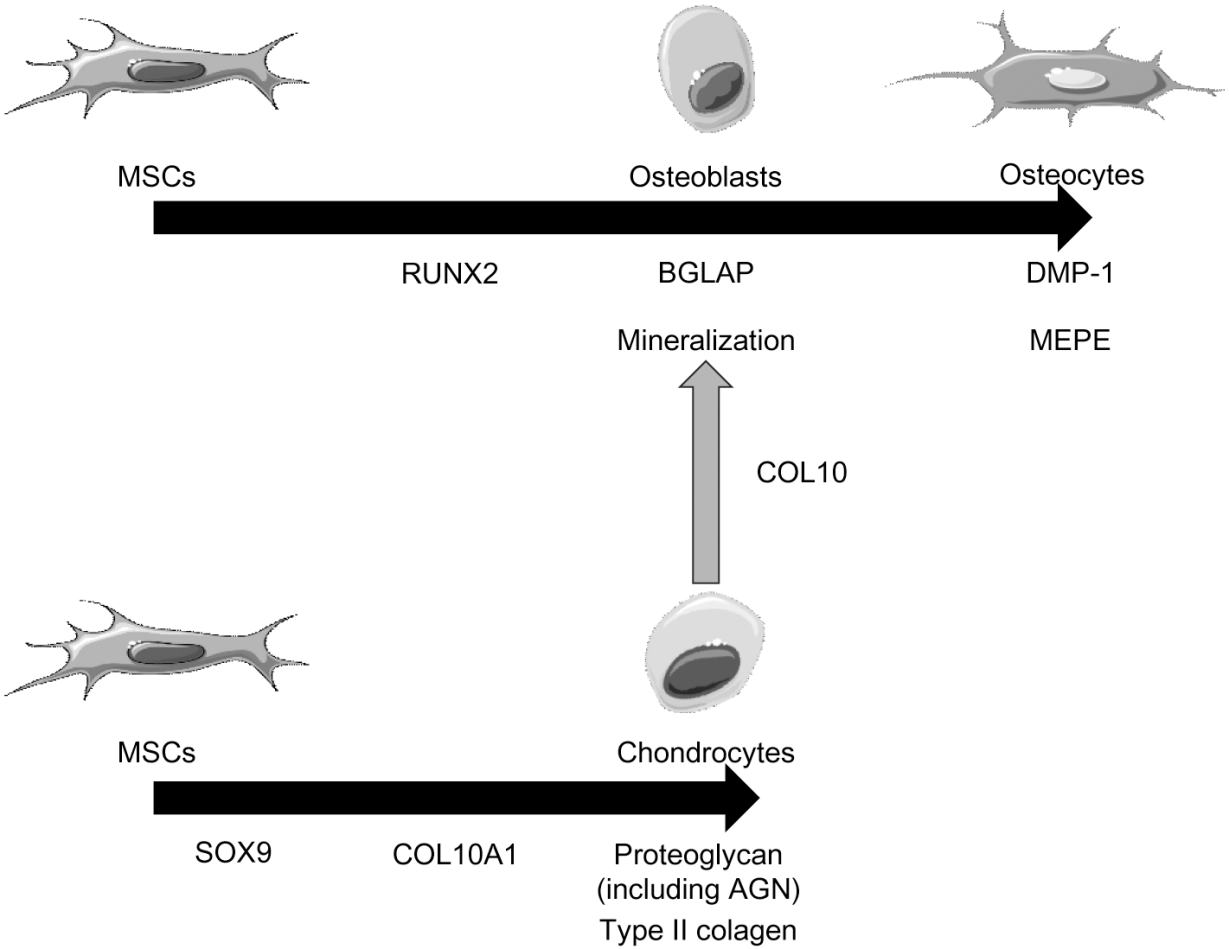
The hierarchy of gene expression in MSCs during differentiaion into osteoblasts, chondrocytes, and osteocytes. MSCs differentiate into osteoblasts or chondrocytes under the regulation of their master regulators, RUNX2 or SOX9, respectively. A part of chondrocytes also differentiate into osteoblasts.

**Fig 3:**
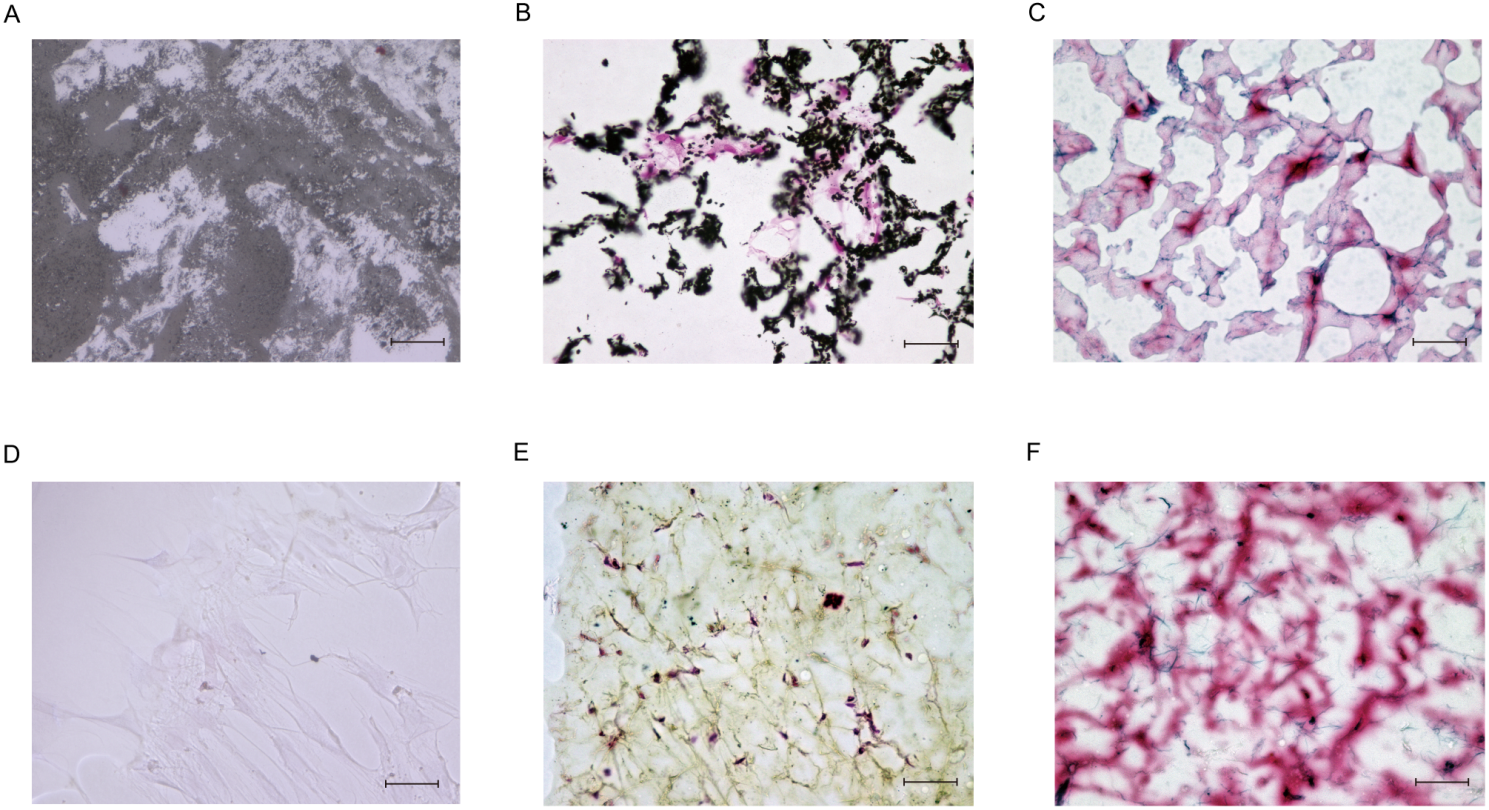
MSCs cultured on nano-fiber plugs produce minerals and proteoglycan upon differentiation stimulation. Healthy MSCs were seeded onto plastic plates (A, D) or injected into the center of the nano-fiber plugs (B, C, E, F), and then cultured in OIM (A-C) or CM (D-F) for 28 days. The samples were then fixed with formalin, and then stained with von Kossa stain (A, B, E) or Safranin O (C, D, F). Representative results of three experiments are shown. Scale bars, 50 μm.

Next, we examined whether MSCs could differentiate into osteoblasts and chondrocytes when cultured on NFs. The hierarchy of gene expression in MSCs during differentiation into osteoblasts, chondrocytes, and osteocytes are shown in Fig 2 [14–16]. When healthy donor-derived MSCs were injected into the NFs and three-dimensionally cultured in OIM for 28 days, calcification was observed in many samples and confirmed by strong von Kossa staining (Fig 3B), as in two-dimensional cultures (Fig 3A). However, no proteoglycan production was observed (Fig 3C). When healthy donor-derived MSCs were injected into the NFs and three-dimensionally cultured in CM for 28 days, marked production of proteoglycan was observed by Safranin O staining (Fig 3F), in comparison to two-dimensionally cultured MSCs (Fig 3D). However, no calcification was observed (Fig 3E). NFs alonewere not positive for both von Kossa and Safranin O staining (data not shown). These results showed that MSCs could undergo calcification and produce cartilage matrix on NFs under appropriate environment.

**Fig 4:**
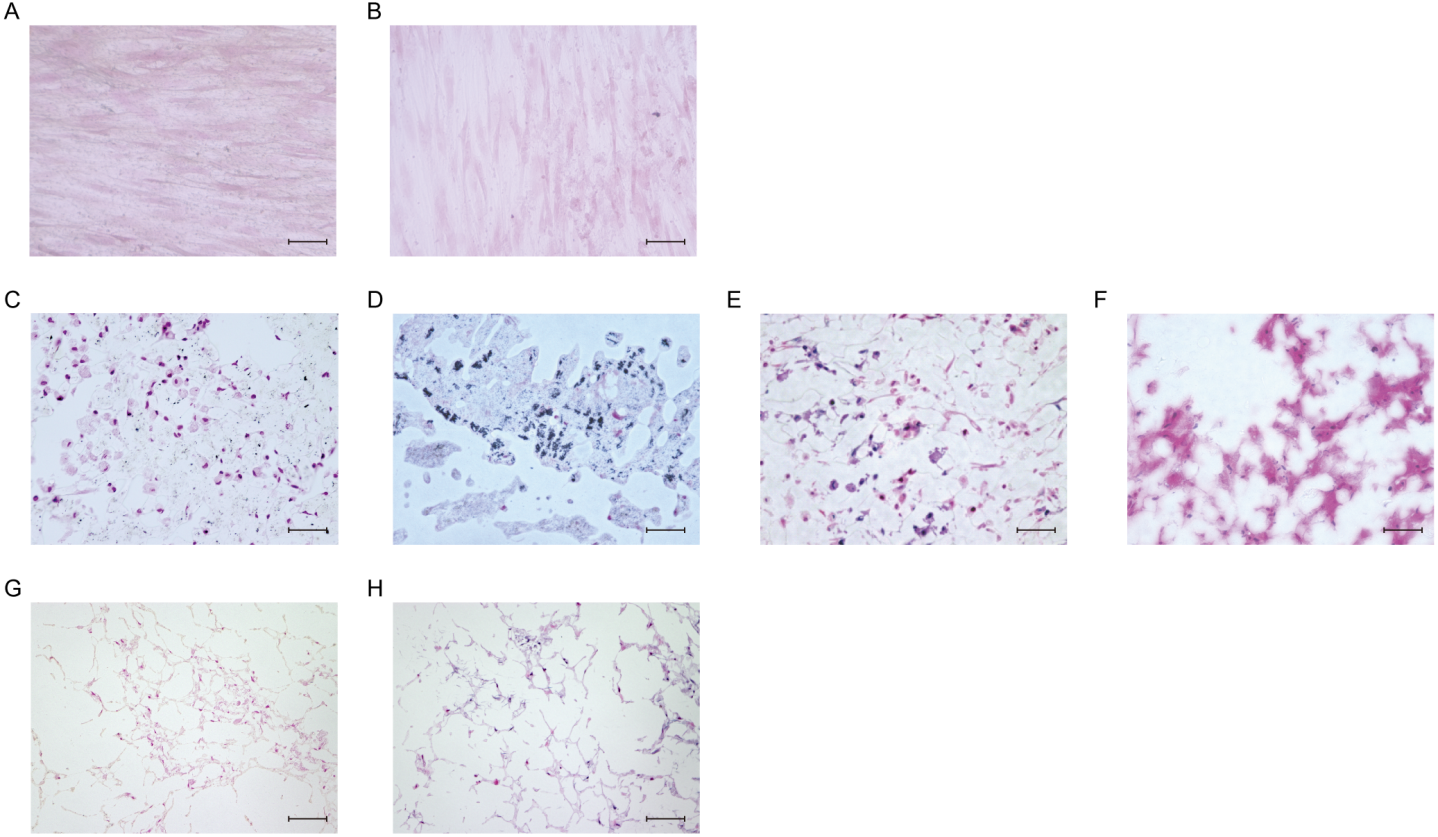
Nano-fiber plugs induce MSCs to produce minerals and proteoglycan. Healthy donor-derived MSCs (A-F) or healthy donor-derived skin fibroblasts (G, H) were seeded onto plastic plates (A, B) or injected into the center of the nano-fiber plugs (C-H), and then cultured in mesenchymal stem cell growth medium for 14 days (C, E), 28 days (A, B, F-H), or 56 days (D). The samples were fixed with formalin, embedded in paraffin, and stained with von Kossa stain (A, C, D, G) or Safranin O (B, E, F, H). Representative results of three experiments are shown. Scale bars, 50 μm.

In our previous experimentswith CIA rats [4], we showed that the immunosuppressive ability of MSCs might be enhanced by seeding on NFs. To explore further NFs function, we evaluated the impact of NFs on the differentiation of MSCs. Even after seeding of healthy donor-derived MSCs in plastic plates and culture in MSCGM, neither calcification nor production of cartilage matrix was observed (Fig 4A and 4B). When healthy donor-derived MSCs were injected into NFs and cultured in MSCGM without differentiation stimuli for up to 56 days, calcification was observed, as visualized by von Kossa staining (Fig 4C and 4D). Moreover, the samples became strongly positive for Safranin O staining on Day 28 (Fig 4E and 4F). By contrast, skin fibroblasts cultured in NFs showed neither proteoglycan production nor calcium deposition (Fig 4G and 4H). These findings suggest that the NFs may have induced MSCs to differentiate into osteoblasts and chondrocytes.

**Fig 5:**
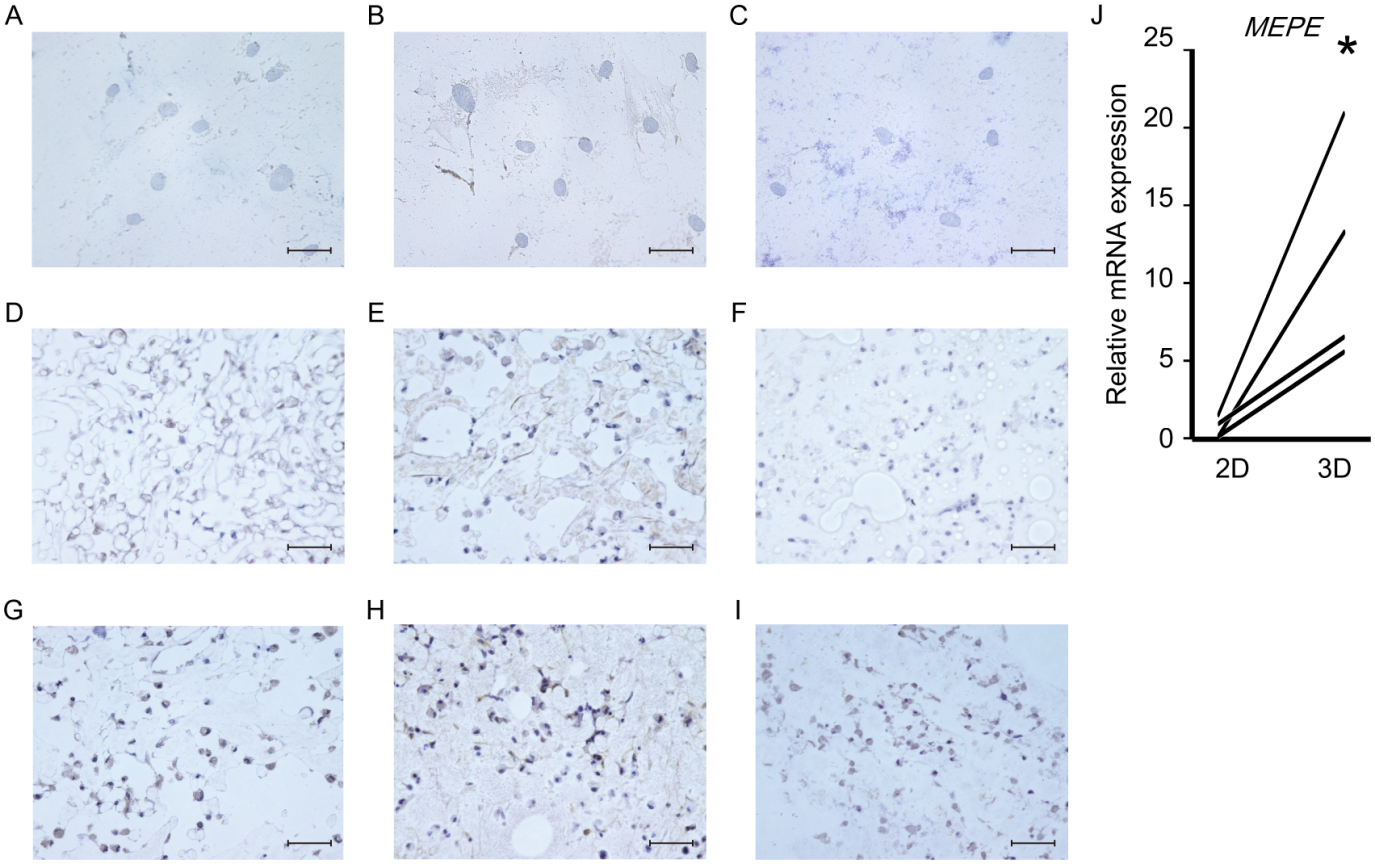
Nano-fiber plugs induce osteogenesis of human MSCs. Healthy donor-derived MSCs were seeded onto plastic plates (A-C) or injected into the nano-fiber plugs (D-I), and then cultured in MSCGM for 1 day (D), 7 days (E, G), 14 days (F, H) and 28 days (A-C, I). Then, the expression levels of RUNX2 (A, D, G), osteocalcin (B, E, H), and DMP-1 (C, F, I) were evaluated immunohistochemically. Representative results of three experiments are shown. (J) Healthy donor-derived MSCs were cultured in MSCGM onto plastic plates (two-dimensional) or nano-fiber plugs (three-dimensional) for 28 days. After RNA extraction, *MEPE* expression in the 4 groups of healthy donor-derived MSCswas evaluated by real-time PCR. *p<0.05 vs. two-dimensional culture by paired *t*-test. Scale bars, 50 μm.

**Fig 6:**
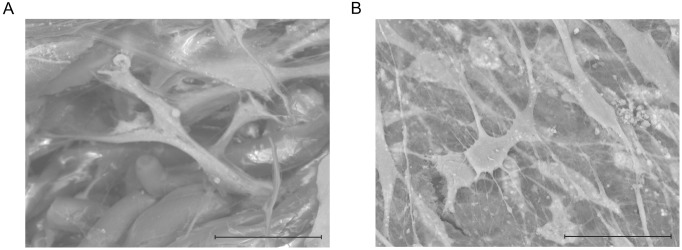
Nano-fiber plugs induce morphological changes in MSCs. Healthy donor-derived MSCs were seeded onto nano-fiber plugs, and then cultured in MSCGM. Scanning electron micrographs taken on Days 7 (A) and 28 (B) are shown. All samples were tested three times, and representative results are shown. Scale bars, 50 μm.

For closer examination, samples were assessed using osteoblast markers (RUNX2 and osteocalcin) and osteocyte markers (DMP-1 and MEPE). First, healthy donor-derived MSCs were two-dimensionally cultured in plastic plates for 28 days, but they expressed neither RUNX2 (Fig 5A), OSTEOCALCIN (Fig 5B), nor DMP-1 (Fig 5C). Next, healthy donor-derived MSCs were injected into NFs and three-dimensionally cultured in MSCGM for 28 days. Immunohistochemical analysis showed that the MSCs became positive for RUNX2 (Fig 5D and 5G), OSTEOCALCIN (Fig 5E and 5H), and DMP-1 (Fig 5F and 5I) expression on Days 7, 14, and 28, respectively, and the level of *MEPE* gene expression increased significantly in the three-dimensionally cultured MSCs (Fig 5J). Moreover, scanning electron microscopy showed that the healthy donor-derived MSCs changed from cells with a spindle-shaped morphology into ones with long dendritic processes over time (Fig 6A and 6B). These results indicate that NFs induce MSC differentiation into osteoblasts and osteocytes.

**Fig 7:**
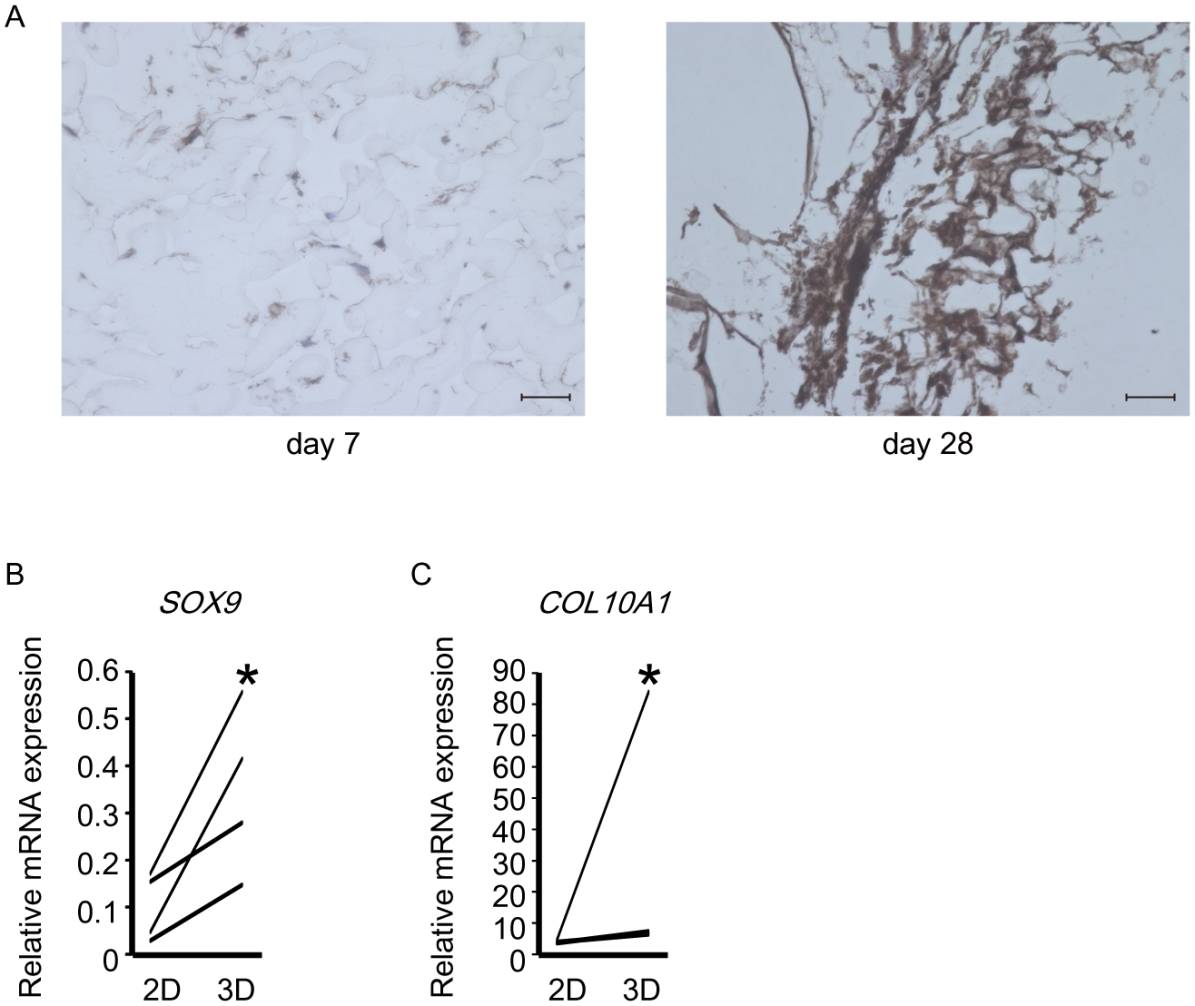
Nano-fiber plugs induce chondrogenesis of human MSCs. (A) Healthy donor-derived MSCs were injected into the center of nano-fiber plugs and cultured in MSCGM for the specified period. The samples were assessed immunohistochemically for the expression of type-II collagen on Days 7 and 28. Representative results of three experiments are shown. Scale bars, 50 μm. (B and C) Human MSCs were cultured in MSCGM onto plastic plates (two-dimensional) or nano-fiber plugs (three-dimensional) for the specified period. After RNA extraction, the expression levels of *SOX9* at day 7 (B) and *COL10A1* at day 28 (C) in the 4 groups of healthy donor-derived MSCswere assessed by real-time PCR. *p<0.05 vs. two-dimensional culture by the paired *t*-test.

Next, we evaluated the ability of MSCs to differentiate into chondrocytes. When healthy donor-derived MSCs were injected into NFs and three-dimensionally cultured in MSCGM for 28 days, marked production of type-II collagen was observed on Day 28, but not Day 7 (Fig 7A). Real-time PCR results showed that the expression levels of *SOX9* (Fig 7B) and *COL10A1* (Fig 7C) were significantly increased in three-dimensionally cultured MSCs at Days 7 and 28, respectively. These results indicated that NFs induced healthy donor-derived MSCs to differentiate into chondrocytes.

**Fig 8:**
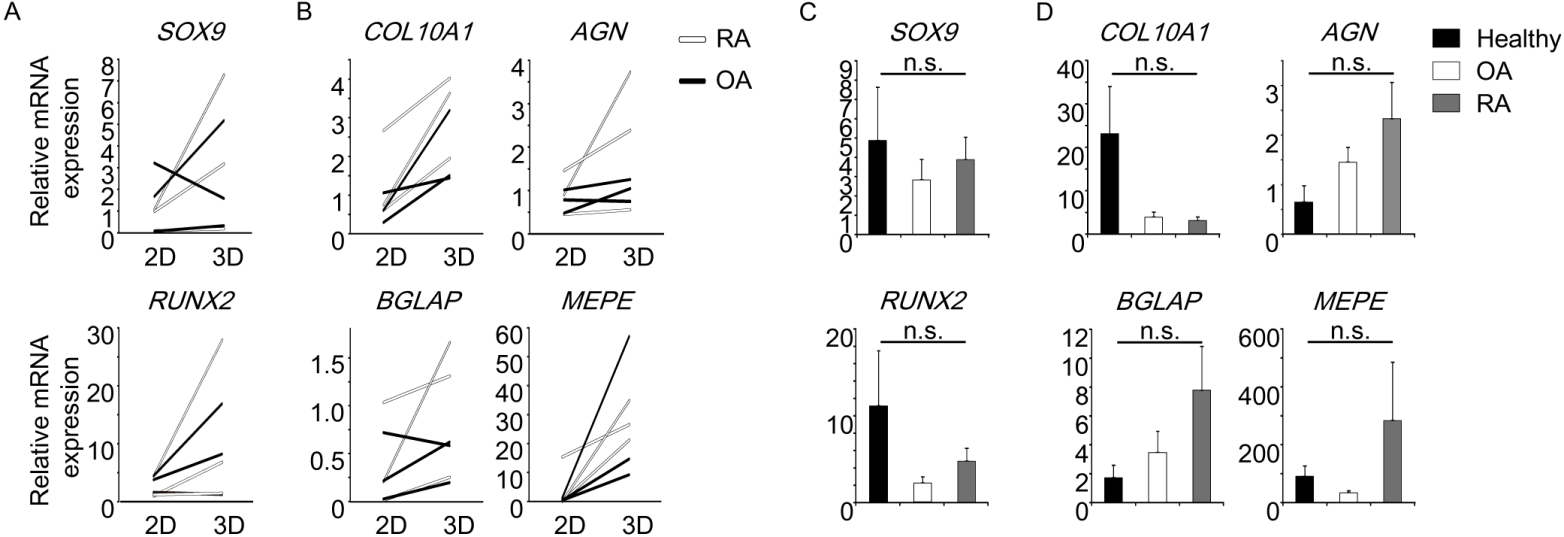
Nano-fiber plugs induce the differentiation of MSCs derived from patients with RA or OA. MSCs derived from patients with RA (n = 3) or OA (n = 3) were cultured in MSCGM onto plastic plates (two-dimensional) or nano-fiber plugs (three-dimensional) for 7 (A) or 28 days (B). After RNA extraction, real-time PCR was performed to evaluate the gene expression level. Each line represents one patient. MSCs from healthy donors (n = 6), RA (n = 3) or OA (n = 3) patients were cultured in NFs for 7 days (C) or 28 days (D), and then analyzed as for their gene expression by real-time PCR. n.s.: not significant by ANOVA.

Similar experiments were performed with patient-derived MSCs. As shown in Fig 8A and 8B, the expression of genes associated with chondrocytes, osteoblasts, and osteocytes was enhanced by three-dimensional culture on NFs in most MSCs derived from patients with RA or OA. Moreover, expression levels of the indicated genes of RA- or OA-derived MSCs were comparable to those of MSCs from healthy donors (Fig 8C and 8D), indicating that NFs also induce osteoblast, chondrocyte and osteocyte differentiation of MSCs derived from healthy donors, as well as patients with RA or OA.

**Fig 9:**
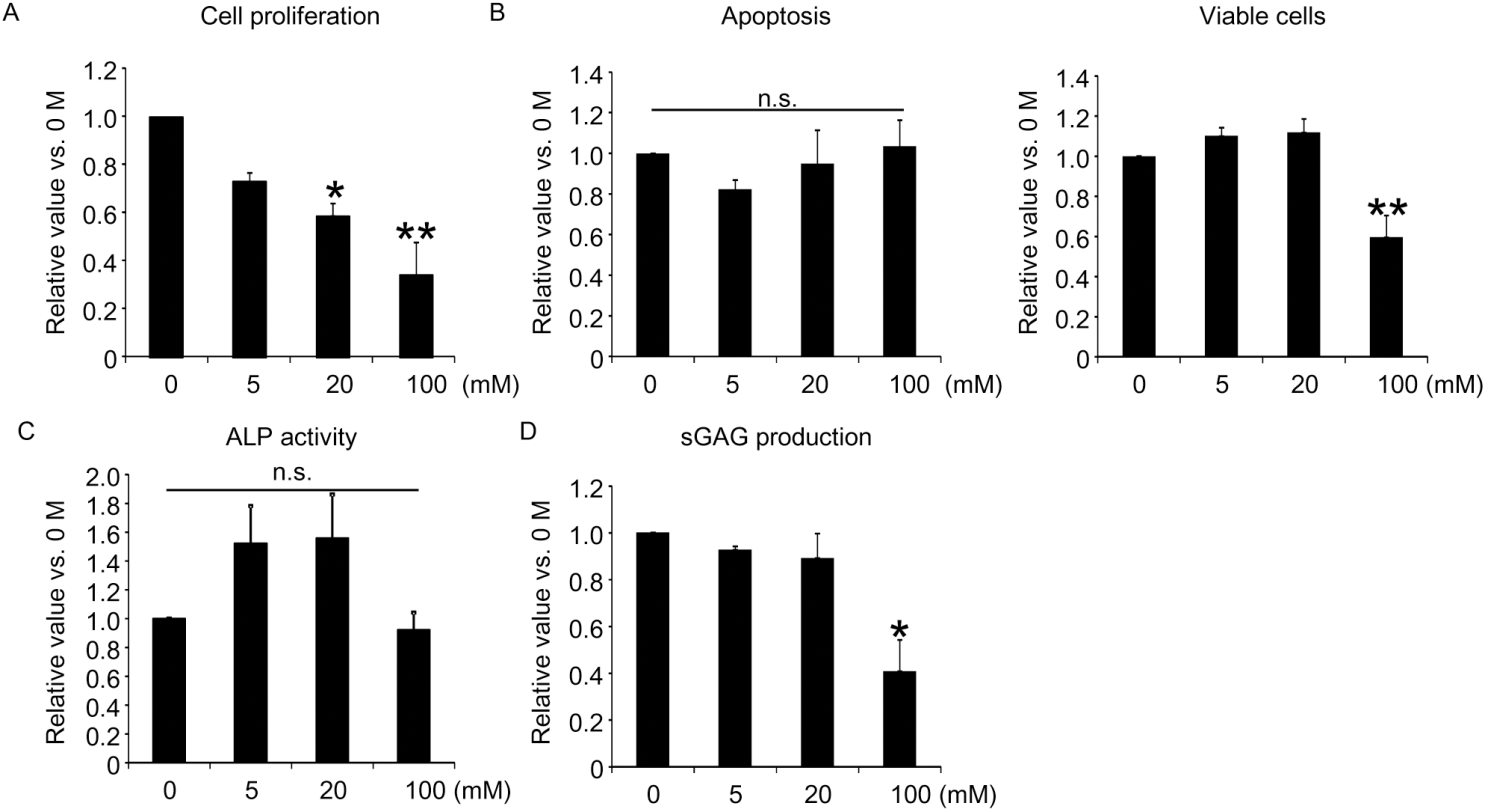
Effects of lactic acid on cell functions of human MSCs. Healthy donor-derived MSCs seeded onto plastic plates were cultured in MSCGM with lactic acid. (A) Proliferative capacity assessed by measurement of ^3^H thymidine uptake after 1 day (n = 3). (B) Propidium iodide-positive apoptotic cells and WST-8-positive viable cells on Day 7 (n = 5). (C) ALP activity on Day 7 (n = 3), and (D) production of sGAG on Day 28 (n = 3). Data are mean±SD. *p<0.05, **p<0.01 vs. 0 mM, by ANOVA with post-hoc Dunnett’s test. n.s.: not significant.

The NFs used in this study release lactic acid as they are gradually hydrolyzed *in vivo* or in culture media. Lactic acid has various bioactivities, such as providing nutrition, tissue protection, and suppression of immune reactions [17]. To investigate the mechanism by which NFs promote the differentiation of MSCs, we evaluated the effects of lactic acid on healthy donor-derived MSCs. It was difficult to measure the concentration of lactic acid where MSCs attached to NFs. Several reports provided the concentrations of lactic acid in non-wounded tissue with 1–2 mM, while wounded tissues contain 5–15 mM of lactic acid or more [18–20]. However, it is possible that lactic acid released from NFs show higher concentration around MSCs than normal tissues. Accordingly, we conducted the next experiments using 0–100 mM of lactic acid. First, the proliferative capacity of MSCs was assessed by measuring ^3^H thymidine uptake; lactic acid suppressed the proliferative capacity of MSCs in a concentration-dependent manner (Fig 9A). Assessment of apoptosis by PI staining showed a decrease in the number of apoptotic cells following the addition of lactic acid (up to 20 mM). WST-8 assay also revealed that the addition of up to 20 mM lactic acid tended to increase the number of viable cells (Fig 9B). These results suggest that lactic acid seems to suppress cellular proliferative capacity and inhibit apoptosis.

Finally, we assessed the effects of lactic acid on MSC differentiation. Treatment of healthy donor-derived MSC with up to 20 mM lactic acid increased ALP activity, indicating that lactic acid contributes to MSCs differentiation into osteoblasts (Fig 9C). However, lactic acid tended to inhibit sGAG production in a concentration-dependent manner (Fig 9D). These results indicate that lactic acid (at 0–20 mM) may promote the survival and differentiation of MSCs into osteoblasts, but inhibit their differentiation into chondrocytes. At high concentration (100 mM), lactic acid inhibits the survival and function of MSCs. Thus, lactic acid has a biphasic concentration-dependent effects on MSCs survival and differentiation.

## Discussion

In this study, we showed that PLGA NFs induced differentiation of MSCs derived from healthy donors and patients with RA or OA into osteoblasts, osteocytes, and chondrocytes.

In the present study, MSCs cultured without any differentiation stimulus differentiated into osteoblasts and chondrocytes in a chaotic manner (Fig 4). However, differentiation could be steered towards a particular cell target by placing MSCs in an appropriate environment. Using a rabbit model of osteochondral defect, Toyokawa et al. [10] reported that transplantation of NFs, identical to that used in the present study, resulted in bone repair and coverage cartilage that was comparable to the surrounding tissue. Because cell transplantation was not performed in their study, it is assumed that the MSCs infiltrated from the surrounding bone marrow and differentiated. Thus, MSCs appeared to differentiate according to the surrounding environment. It can be expected that MSCs transplanted *in vivo* with NFs will not differentiate in a random fashion, but rather differentiate according to the surrounding tissue. On the other hand, inflammatory circumstance may also affect differentiation of MSCs transplanted with NFs. As we have previously reported that certain inflammatory cytokines induce differentiation of MSCs into osteoblasts [8] and inhibit differentiation into chondrocytes [21]. These may raise concerns that transplantation of MSCs into a joint with residual inflammation is associated with the risk of heterotopic ossification. Contrarily, implanted MSCs are possible to suppress arthritis and bone damage by their immunosuppressive effect [6] and have ability to suppress osteoclast differentiation [7]. However, we consider that it is preferable in clinical practice to apply these cells after sufficient control of inflammation by drugs and other therapeutic measures, to minimize the risk of therapeutic failure.

While the use of different PLGA scaffolds have been reported, PLGA scaffolds promote MSC proliferation [22] and MSC differentiation into osteoblasts [23] under osteogenic culture condition. Scaffolds containing TGF-beta [24] and stromal cell derived factor-1α [25] induced cartilage regeneration in animal models. However, our study is the first to show that the NFs themselves induce the differentiation of MSCs not only into osteoblasts, but also into osteocytes and chondrocytes. Moreover, treatment with the combination of cytokines and other factors is commonly used to improve the function of scaffolds. However, the NFs used in our study possess a feature not observed in other materials; namely, induction of the respective cell differentiation above without the addition of cytokines.

What are the mechanisms by which PLGA scaffolds induce proliferation of MSCs and differentiation into osteoblasts? While the exact mechanisms are unknown at present, it is thought that scaffolds can enhance the adhesive capacity of MSCs [22, 26] or that the 3-dimensional culture is more physiological and increases the cell potential. The NFs used in our study could have induced differentiation of MSCs through the following mechanisms: 1) lactic acid present in the NFs may have induced survival and differentiation of MSCs into osteoblasts and chondrocytes (Fig 9), or 2) the NFs may have enhanced secretion of soluble factors from MSCs. Regarding the first mechanism above, the data obtained in our study showed that specific concentrations of lactic acid inhibited both proliferation and apoptosis of MSCs, increased the number of viable cells, and induced ALP activity. However, these effects were small and, furthermore, glycolic acid also present in the NFs inhibited MSC proliferation, induced apoptosis, and reduced the number of viable cells (data not shown). Thus, although the data showed that lactic acid contained in the NFs might have contributed to the differentiation of MSCs, its contribution seems to be limited. Regarding the second mechanism above, the results of our previous study indicated increased TGF-beta production in MSCs seeded on NFs [4]. PLGA scaffolds cannot alone induce chondrocyte differentiation, and other methods have been applied [24]. Because the NFs used here may be superior in induction of TGF-beta production or better suited for capture of produced TGF-beta, we hypothesize that the NFs tend to favor chondrocyte differentiation. In fact, the surface area of the NFs increased during culture, which may reflect increased amount of protein absorbed. Further studies are needed to investigate the above mechanisms in order to develop NFs that can induce differentiation of MSCs into osteoblasts, osteocytes, and chondrocytes.

The NFs used in the present study contained glycolic acid solution to control the rate of *in vivo* degradation. This solution, which is highly acidic, inhibited proliferation of MSCs and induced apoptosis (data not shown). PLGA scaffolds are widely used in clinical practice, and there is little concern regarding their safety. However, animal experiments and other studies are required to examine whether MSCs seeded on the NFs used here can differentiate *in vivo*. According to a report by Toyokawa et al. [10], their success in osteochondral repair appears to be due to the use of endogenous cells without transplantation of exogenous cells. However, MSCs derived from RA or OA patients with joint destruction may show impaired function. For future clinical applications, NFs may need to be optimized with patient-derived MSCs before transplantation.

The ultimate aim of the present study was to determine whether transplantation of MSCs/NFs actually leads to joint repair. Regarding this, our study includes a limitation that *in vivo* experiments are lacking. However, in a study of NFs transplantation into a rabbit model of osteochondral defect, complete repair of osteochondral defects was noted following cellular infiltration from the surrounding areas [10]. The same study also showed possible bone and cartilage repair following transplantation of NF alone. Namely, NFs are expected to repair damaged joint by itself. On the other hand, although our data suggested the comparable differentiation abilities of RA- or OA-derived MSCs combined with NFs to healthy donor-derived MSC (Fig 8C and 8D), many RA or OA patients with advanced joint destruction are elderly, and patient-derived MSCs may be either insufficient in number or inferior in function. Also, the safety of allotransplantation of MSCs has been demonstrated by several clinical studies [6, 27, 28]. Taken together, both auto- and allo- transplantation of ex *vivo* expanded MSCs combined with NFs s possible for the new treatment strategy of damaged joints.

## Conclusion

We have demonstrated in the present study that PLGA NFs induce MSCs derived from healthy donors and patients with RA or OA to differentiate into osteoblasts, osteocytes, and chondrocytes, which mimicked the procedures of endochondral ossification. Regarding immunosuppressive activities, and ability to inhibit differentiation of monocytes into osteoclasts by MSCs, MSCs combined with NFs can be potentially useful for the treatment of joint diseases.
